# On Ankylosis or Stiff-Joint; a Practical Treatise on the Contractions and Deformities Resulting from Diseases of the Joints

**Published:** 1844-10

**Authors:** 


### ART. VII.

1. *On Ankylosis or Stiff-joint; a Practical Treatise on the Contractions and Deformities resulting from Diseases of the Joints.* By W. J. LITTLE, M.D.—*London*, 1843. 8vo, pp. 146.

2. *Traité Pratique du Pied-bot, de la fausseAnkylose du Genou, et du Torticolis.* Par V. DUVAL.—*Paris*, 1843.

*A Practical Treatise on Club-foot, False Anchylosis of the Knee, and Wry-neck.* By V. DUVAL.—*Paris*, 1843. Second Edition, 8vo, pp. 542.

3. *Du Traitement des fausses Ankyloses et de la Contracture des Membres par la Compression, aidée de l'Extension, sans l'Emploi de la Tenotomie, avec quelques Reflexions sur ce dernier mode opératoire.* Par M. DANCEL.—*Paris*, 1843.

*On the Treatment of False Anchylosis and Contraction of the Limbs by Compression aided with Extension, without the performance of Tenotomy, with some Reflections on the latter operation.* By M. DANCEL.—*Paris*, 1843. 8vo, pp. 76.

IN several former Numbers of this Journal, (XVI, Oct. 1839, XXV, Jan. 1842, XXVII, July 1842,) the general principles and most of the special applications of subcutaneous tenotomy have been fully examined; but we have not yet had an opportunity of sufficiently considering the employment of that method in the treatment of anchylosis, and take advantage of the present occasion to draw attention to that most important subject.

It would be an unnecessary waste of time and space to dwell on the inefficiency of all the older methods recommended for the treatment of confirmed false anchylosis. Occasionally no doubt, when the affection existed but in a slight degree, it was alleviated or even removed, but as a general rule, confirmed anchylosis was abandoned as incurable, and when the knee was implicated, not unfrequently led to amputation of the limb. Scarcely seven years have elapsed since M. Duval first applied tenotomy to the cure of this intractable deformity, and so brilliant has been the progress of this branch of surgery within that limited period, that Dr. Little has fallen into little if any exaggeration in saying that few cases of false anchylosis are incapable of alleviation or cure. We feel it to be our duty therefore to notice the works enumerated at the head of this article, (especially those of Dr. Little, and of M. Duval,) as briefly as is consistent with clearness, for "although," to use Dr. Little's words, "the method in question has now been frequently practised in this country, and occasionally a successful case has been announced in the medical periodicals, no complete exposition of the forms of anchylosis in which it is especially useful, or the details necessary to enable the competent practitioner successfully to apply it, has been hitherto published." (p. 1.) For the sake of convenience and brevity, we shall take false angular anchylosis of the knee-joint as the type of the affection, and append any necessary observations relative to the deformity as it occurs in other joints.

Long-continued inflammation, no matter what its origin, is universally admitted to be the exciting cause of anchylosis in the vast majority of cases, but it is still a disputed point whether protracted immobility of a limb can produce anchylosis independent of an antecedent disease in the contracted joint. M. Duval, with Petit, Hunter, Boyer, &c., admits mere immobility to be a cause of anchylosis, but Dr. Little rejects "the simple *state* of *rest* or *disuse*, as a cause of true anchylosis," (p. 11, Note,) and by implication rejects it as a cause of *false* anchylosis also, as he does not enumerate it in his table of the causes of false anchylosis, (pp. 12-13;) and at p. 2, states that "inflammation is the cause of each variety and grade of anchylosis." Dr. Little supports this opinion, which has been previously advanced by Cruveilhier, Kunholtz, and other authorities, by an appeal to the fact that, in the most aggravated cases of congenital club-foot of thirty or forty years' standing, the tarsal joints, some of which have probably been motionless for many years, may be restored to their natural function, and by conjecturing that Cloquet was deceived in supposing that he had witnessed bony anchylosis in the joints of individuals who had long remained motionless in consequence of paralysis; on this subject we shall merely observe that though the two remarkable cases published by Cruveilhier and Kunholtz, in which complete immobility of the lower jaw, for the respective periods of sixty and eighty-three years, failed to cause anchylosis, come in aid of Dr. Little's argument, yet such cases merely prove that anchylosis is not a constant, probably not a frequent effect of continued rest, but they do not warrant us in setting aside the positive testimony of so competent an observer as M. Cloquet, on conjectural grounds, especially when the more recent investigations of M. Teissier (Gazette Méd. de Paris, t. ix, pp. 609-26,) go so directly to prove that a long-continued state of rest may cause vascularity of the synovial membrane, with formation of false membranes, absorption of the cartilages, and finally true anchylosis.

In the vast majority of cases, however, anchylosis, as already said, originates in some inflammatory disease of a joint; but how is it that such inflammatory affection in the first instance so frequently causes flexion of the joint, and that the flexion ultimately becomes permanent? M. Duval adopts the usual explanation that the inflammation in or about the joint, excites the irritability of the flexor muscles, and consequently the patient relaxes them by bending the knee. To this Dr. Little objects that there is no reason why the extensor muscles should not be as much irritated to preternatural action as the flexors, and he explains the preference given in most cases to the bent position of the knee: 1st, by the fact that *most* of the muscles of the extremity are then relaxed, because though some of the extensor fibres may then be rendered tense, yet as the thigh is also flexed, the rectus femoris being attached to the pelvis, is relaxed; and 2dly, by the circumstances, that the amplitude of the synovial sac being greater during flexion than extension, its fluid contents exert less pressure on the inflamed tissues. (pp. 4-5.) We believe Dr. Little's views on this subject are correct at least in part, but they have been anticipated by M. Bonnet, (Gaz. Méd. de Paris, 1841, Nos. xlvi and xlvii,) who points out some other circumstances which more constantly tend to cause flexion of the joint, the chief of which is simply the necessity experienced of keeping the limb as motionless as possible, which can only be done with facility when the limb is extended so long as the patient lies on his back, for the moment he turns even partially on the side, the entire body has an insufficient basis of support and steadiness of posture, can only be maintained without a constant and fatiguing exertion by flexing both the leg and the thigh, by assuming in fact the position which we almost constantly adopt in perfect health when lying on the side in bed. That the capacity of the synovial membrane is greatest when the joint is flexed has been proved by M. J. Guerin, and by M. Bonnet, who has, however, also shown that probably this circumstance has no influence on the position of the limb so long as the quantity of fluid contained in the joint is small: if indeed the accumulation is sufficient to produce extreme distension, flexion of the joint follows as a matter of physical necessity, for even in the dead subject if fluid is forcibly injected into the cavity of the knee-joint, the leg becomes bent to a right angle with the thigh.

But though the muscles it would appear are not the primary agents in flexing a diseased joint, they unquestionably exert a most important influence in keeping it permanently flexed after the bent position has been maintained for a certain time, and when perhaps the original disease may have completely subsided. No doubt any alteration in the shape or relative position of the articular extremities of the bones, or the effects of inflammation, such as the effusion of lymph, and the thickening of the structures while the joint was flexed, tend to keep it in that posture, but whenever the deformity has existed for any considerable period, contraction of the flexor muscles usually constitutes the chief obstacle to straightening the limb. This contraction is usually attributed merely to the continued operation of the organic contractibility of muscular fibre consequent on the permanent approximation of the fixed extremities of the relaxed muscles; but Dr. Little maintains that "after the lapse of some time, the shortening from this cause attains the maximum, and is followed by a change in the structure of the muscular fibre, which I have named structural shortening," (p. 7,) and which consists in interstitial shortening of the muscle in consequence of its molecules being renewed while it is contracted, so that "the muscular fibres become permanently shortened and inelastic; and provided a period sufficient for the complete renewal of their integrant parts has elapsed, they are necessarily redeposited shorter, to accommodate them to the altered relation of the surrounding parts." (p. 8.) This latter change, Dr. Little says, is shown by observation, as might be expected, to occur more rapidly in children than in adults, and the relative shortness of the muscles is further increased in early life, because "the bones, gradually advancing towards their full development, are elongated, the disproportion between them and the contracted muscles is augmented, and the deformity of the limb necessarily aggravated." (p. 8.) Thence it is that "in a favorable case of false anchylosis in an adult, we may succeed in effectually straightening the limb after the lapse of five years, (without tenotomy;) but it is rarely possible in a child, unless of very lax fibre, permanently to relieve by mechanical means, a severe contraction of similar duration." (p. 31.)

The diagnosis of anchylosis from other affections of the joints can rarely indeed be attended with any difficulty. Dr. Little says a mistake is impossible, and we should have been inclined to think so too, were it not for the case mentioned by Sanson (Dict. de Méd. et Chirurg. Prat., art. Ankylose,) of a child whose hip-joint, though affected with coxalgia, was supposed by several eminent practitioners to be anchylosed, the thigh being so immoveably flexed on the pelvis that when the patient was raised by the limb, the weight of the body failed to cause any motion in the hip-joint; on diverting the child's attention, however, the limb could be momentarily extended, but directly this was perceived, the thigh was again rigidly fixed in its former position. But though it is almost uniformly very easy to detect the existence of anchylosis, it is sometimes extremely difficult to determine whether the anchylosis is true or false. With respect to the diagnostic signs between those affections, Dr. Little attaches little importance to the statement that in true anchylosis the patient apprehends no pain from an examination of the joint, but that the reverse is the case in false anchylosis. A sense of pain or tightness produced on the flexed side of the limb, by attempts to straighten the joint, often indicates incomplete anchylosis. "But," in Dr. Little's opinion, "a much more delicate test is the production of pain on the opposite side," (p. 28,) though in two cases of bony union of the patella to the femur, he found this test fallacious. The condition of the muscles on attempting to straighten an anchylosed joint, is an important index of the nature of the case, for in false anchylosis, even when no motion is visible, "an alternate tension and relaxation of the muscles may be produced by forcible attempts made to straighten and bend the knee," (p. 27,) while no such tension of the muscles can be mechanically produced in true anchylosis. Dr. Little, however, warns us that "muscular tension is a symptom of anchylosis which may occasion an erroneous diagnosis," (p. 27,) as the patient while the limb is being examined, is apt to throw the muscles into voluntary action, which may be mistaken for tension consequent on the attempt to straighten the joint; and in order to avoid this source of error, "the practitioner will observe whether the muscular tension be synchronous with his manual attempts to move the joint," (p. 27,) and will take care that the patient's mind is diverted during the examination. (p. 46.) Dr. Little very candidly states, (pp. 123-4, Note,) that he fruitlessly performed tenotomy in one case before he "*discovered* the source of error in diagnosis" now adverted to. It is, however, right to say that this discovery does not appertain to Dr. Little; it was long since distinctly stated by M. Duval, who recommends us, in order to avoid being thereby misled, to place the patient on a sofa, and measure the space between the ischium and heel, first while the limb is at rest, and then when it is extended, the pelvis having been firmly fixed. If the two measurements are equal, the anchylosis is complete, if the latter is the longer, the case is one of false anchylosis.

Dr. Little and M. Duval, agree that the prognosis of anchylosis is in the vast majority of cases favorable, inasmuch as true anchylosis occurs but very rarely, and false anchylosis is almost uniformly remediable, whatever may be its duration. When the disease indeed has existed for a very long period, the treatment is both tedious and difficult, and tests both the resources of the surgeon and endurance of the patient, but still both authors agree that success more or less complete may be calculated on, as they have each effected cures where the deformity existed for periods varying from ten to twenty years, and in one instance Dr. Little achieved a cure after twenty-six years. But though so far agreed, Dr. Little and M. Duval differ very materially as to the *amount* of success obtainable at least in aggravated cases. Dr. Little teaches that the result is generally perfect, or as near thereto as may be, that not only can the limb be straightened, but the motions of the joint can in most cases be restored; but M. Duval, on the contrary, expressly and repeatedly warns us to be most guarded in promising the patient more than that the limb shall be straightened, as "most frequently when the leg is extended on the thigh, the limb remains in that state, that is, the leg cannot be flexed, the patient walks with a stiff joint," (p. 367,) and a little further he adds, "unfortunately, *six patients out of ten* have *lost for ever* the faculty of *flexing* the leg after it has been extended, although the intermediate substance has been perfectly developed between the extremities of the divided tendons. The same thing it is true occurs when a limb has been straightened by mechanical means, without preliminary subcutaneous section of the tendons. When the leg is extended it cannot be flexed, *it remains stiff for ever.*" (p. 368.) But if M. Duval differs in this respect from Dr. Little, we must add that he also differs from himself, for in another passage we find him saying, "the *vast majority* of patients on whom I have operated, already exceeding 150 in number, have been able after the complete extension of the previously flexed leg, to perform *a part or the whole of the natural motions of the knee*," (p. 371;) but in not one of the numerous cases detailed in his work, does he state whether the joint enjoyed any degree of motion after the treatment was concluded. As regards the facility of extending the limb, M. Duval states that, generally speaking, the more moveable the joint, the more favorable is the prognosis in this respect, but that the condition of the articular surfaces is a more important consideration, for if the tibia is either much rotated or displaced backwards on the femur, it may be very difficult to effect extension, though a considerable degree of motion exists in the joint, while an almost immoveable joint may be easily straightened if the relations of the articular surfaces are not disturbed. (pp. 368-9.) M. Duval says that the difficulty of cure generally increases with the age of the patient, and the duration of the disease; we have already seen that so far as the mere influence of age is concerned, Dr. Little comes to the directly opposite conclusion. As respects the *cause* of the affection, Dr. Little considers that rheumatic false anchylosis demands a very "cautious prognosis," for though there is little absolute disorganization of the structures, and still less change in the form of the knee, yet the tissues are "often soldered as it were into a mass by the exudation of plastic matters." (pp. 97-8.) Anchylosis after white swelling, on the contrary, though peculiarly prone to be accompanied with great deformity of the joint, "constitutes the most favorable form for division of tendons, the subsequent mechanical treatment being more easy, and the restoration of the limb more complete than in anchylosis from rheumatism, or from extensive suppuration from phlegmon." (p. 115.) We may here mention that though as a general rule, true anchylosis must be deemed incurable, Dr. Little entertains "no doubt of the capability, in some instances, of restoring motion in an articulation between the surfaces of which the deposit of osseous particles has already commenced," (p. 33:) this opinion is founded on two cases given at pp. 92-4, which however, in our opinion, are anything but conclusive; though we must not be understood to deny the possibility of overcoming partial bony anchylosis,—for we are, on the contrary, convinced that it may be occasionally practicable.

Dr. Little and M. Duval agree that friction, manipulation, mechanical extension, &c., are inefficient except in slight cases of anchylosis, unattended with permanent shortening of the flexor muscles, and that when such shortening exists, attempts at mechanical elongation will not only be fruitless, but liable to excite serious mischief in the joint, unless muscular resistance is previously neutralised by the performance of tenotomy. Dr. Little divides the treatment of anchylosis, "by the combined surgical and mechanical method," (p. 32,) into three stages: 1st. The surgical operation, (tenotomy.) 2. "The mechanical reduction of the anchylosis." 3. "The period employed in restoring freedom of movement by passive and active exercises, frictions, manipulations, baths, &c." (p. 32.) M. Duval seems to limit the treatment to the first two of those stages, for neither in the reports of his cases nor elsewhere, does he allude to any treatment for restoring the mobility of the joint after the limb has been straightened. This accounts for the different statements of those authors respecting the amount of benefit derivable from treatment, and also for the great difference in the length of time consumed by them respectively in the treatment. M. Duval says the average period necessary to obtain a "*complete* cure" is six weeks, and seems to fix the maximum time at three or four months, which longer period moreover he has only found necessary in five or six cases. (p. 446.)

Dr. Little, on the contrary, says "the average length of time occupied in the straightening and reduction of the displacement of the articular surfaces after the operation in adults is two months; in children the success is more rapid. The process of restoring mobility . . . . . is seldom completed in severe cases, within a period varying from three to six months," (p. 57,) and occasionally twelve months may elapse "before the patient can be considered fully restored."

Dr. Little considers tenotomy inadmissible, "prior to the complete subsidence of the primary articular disease . . . . until at least two or three years have elapsed since the subsidence of active disease," (p. 117;) and having in one case neglected this rule, and performed the operation at an earlier period, the strumous disease was again excited in the joint. We certainly think that to delay two or three years is to wait a period at least double what is necessary, but otherwise the practice here recommended, is we believe so entirely consonant with the views of surgeons in these countries, that we should not have adverted to this point, if it was not for the opposite doctrine inculcated by M. Duval, who, though he at first carefully avoided operating until the disease of the joint had disappeared, has been long in the habit of performing tenotomy, and straightening the limb during the sub-inflammatory period of white swelling. M. Duval appeals to theoretical considerations and clinical facts in support of this practice. He conceives that maintaining the natural position of the limb is as essential in white swelling and in all chronic inflammations in or about joints, as in fractures, (p. 434;) that straightening a joint must act favorably on any disease under which it may suffer by bringing the greatest possible extent of the articular surfaces into apposition, "and so preventing the contact of angles and obviating the resulting painful pressure," (p. 433;) that while the vicious position of the parts continues, a mechanical cause exists to keep up the disease which antagonises every therapeutic means employed, so that the local disease continues even though the constitution may be improved. (p. 435.) Influenced by such considerations, M. Duval was induced to straighten diseased knees after having divided the ham-string tendons, and states that he has always succeeded in obtaining extension in a period varying from one to two months, not merely without the occurrence of any unpleasant symptoms, but with the happy effect of favorably modifying (p. 436) and ultimately curing the disease of the articulation, (pp. 433-8,) not to mention that the occurrence of anchylosis, of partial luxation of the tibia backwards, &c. is also obviated, and that the more certainly the earlier the diseased joint is operated on. In fine, M. Duval says:

"Supported by numerous facts, I feel warranted in maintaining the following doctrine: Pain, inflammation, alteration of parts, whether inter-capsular or extra-capsular, alteration of the integuments, inflammatory or œdematous swelling, numerous cicatrices, suppurating ulcers—all circumstances, which seem to be so many contraindications, should not arrest the operator, and are, on the contrary, indications for his interference. The prejudices which induce practitioners to remain quiescent under such circumstances must yield to facts.

"Tenotomy, then, offers great and valuable resources to surgeons in the treatment of those tedious and cruel diseases which, having assumed a chronic form, often resist the most prudent treatment, even when the constitution is not impaired." (pp. 438-9.)

M. Duval does not inculcate this practice as an universal rule; he says he is aware that there are many exceptions; we do not however find any specification of the circumstances which constitute those exceptions; unless, indeed, the existence of tolerably acute inflammation. Several French surgeons have adopted M. Duval's views; for example, while penning these lines, a clinical lecture on the indications for performing Tenotomy, by M. Guersant, surgeon to the Hôpital des Enfans, at Paris, has fallen under our notice, (Gazette de Hôp. de Paris, July 4th, 1844,) in which he lays it down as a rule that "in white swelling of the knee it is almost always advantageous to practice tenotomy the moment circumstances are tolerably favorable for its performance; that is to say, when the tumour is not *extremely* painful, when the inflammatory symptoms *begin* to diminish in intensity." But though we must presume that M. Guersant speaks from experience, and though M. Duval gives several cases in which joints affected with white swelling was straightened with a favorable issue, (pp. 417-39-41,) we cannot but regard the practice as most perilous, and should require no small mass of evidence of its innocence and utility to induce us to adopt it.

M. Duval describes, at considerable length, and with more minuteness than Dr. Little, the details of the surgical and subsequent mechanical treatment of false anchylosis of the knee; we shall refer to a few points respecting which he and Dr. Little disagree, or which the latter author does not notice. M. Duval does not appear to have ever found it necessary to divide any structures save the ham-string tendons, and in one instance only the tendon of the gracilis muscle; occasionally he finds it sufficient to divide the biceps alone, especially when the anchylosis is complicated with deviation of the knee inwards; though sometimes, after the lapse of fourteen or fifteen days, when extension of the leg has made considerable progress, the tendons of the inner ham-strings become prominent and must be divided. This secondary operation we think should always be avoided if possible by dividing, in the first instance, every structure which appeared to contribute in maintaining the deformity. Dr. Little, in common we believe with most operators, has to divide occasionally many more structures than those mentioned by M. Duval; such as the interior fibres of the vastus externus muscles, bands of fascia in the ham and on the outer side of the thigh, and the tendon of the sartorius muscle. Dr. Little, of course, warns us against injuring any important nerve; such as the peroneal, which was severed along with the biceps tendon in a patient whom he subsequently had an opportunity of seeing; but he insists on the necessity of "sometimes" dividing "the more superficial of the nerves which supply the gastrocnemii, or traverse its surface." (p. 52.)

"The section of the nervous filaments in question can only be deemed essential after having divided the muscular structures and fasciæ, when the operator will sometimes discover one or more cords rendered tense, on endeavouring gently to extend the limb. If unaware of their nature, before their section he might suppose them to be fibrous bands; but the peculiar pain complained of by the patient indicates their character. An experience founded on the performance of the operation in numerous cases, and the management of the subsequent mechanical treatment, has shown me the propriety of not leaving these filaments undivided. If stretched during the after treatment, very severe pains may be experienced during weeks or even months. In short, I am convinced that much suffering is spared by the section: the filaments reunite, and recovery from the anchylosis ensues without neuralgia, and without numbness or diminished power in the muscles." (pp. 52-3.)

In dividing the ham-string tendons, M. Duval introduces the knife beneath the tendon and divides it from before backwards or towards the skin: Dr. Little gives no direction on this head, but we presume he follows Bouvier's method of insinuating the tenotome between the skin and the tendon, and cutting from the superficial towards the deep parts, which we think is decidedly the preferable mode. Our readers are aware that considerable difference of opinion and practice exists as to the period when extension should commence after tenotomy has been performed. M. Duval is an advocate for immediate extension; directly after the operation he applies an apparatus to maintain the amount of extension thereby obtained: Dr. Little, on the contrary, and in our opinion most correctly, thinks it most important not to commence the mechanical extension till the punctures are completely cicatrized, which, according to M. Duval, usually occurs in two days; according to Dr. Little, not until the third or fourth (p. 53), or rather, we are told (p. 116), the fourth or fifth day. On M. Duval's own showing, it appears, we think, that the practice of immediate extension is injudicious, as he speaks of pain in the joint requiring intermission of the treatment, &c., as a frequent occurrence. In the only instance in Dr. Little's practice in which the operation was followed by an abscess in the ham, Dr. Little is in doubt whether the suppuration was due to the extension having been commenced previous to the complete cicatrization of the punctures, or to the circumstance that the biceps tendon having been divided at two adjacent points, the portion of the tendon included between the two sections lost its vitality from being insufficiently supplied with blood, and therefore acted as a foreign body. Be this as it may, whenever a tendon requires to be divided a second time, Dr. Little recommends that the second operation should be postponed for two or three weeks in order to allow complete vascular union to be established in the part first divided. (p. 116.)

The mode of conducting extension after the division of the tendons is a matter of great importance, and must vary with the circumstances of the case. Without a very minute description, indeed without figures of the requisite machinery, it would be impossible to enter satisfactorily into details on this head, and we shall limit ourselves to a few general observations. When anchylosis is unaccompanied with displacement of the bones, a very simple apparatus, occasionally even a common splint bandaged on the posterior surface of the joint, may be sufficient to straighten the limb. But when the head of the tibia is rotated, or partially luxated backwards on the condyles of the femur, it is essential to employ an apparatus calculated to remedy such displacement; for not only would the deformity otherwise be very imperfectly removed, but Dr. Little has "ascertained from repeated experience, that when attention has been directed to the removal of the abnormal flexion only, an increased displacement of the head of the tibia sometimes resulted." (p. 541.) And in one case he "found the luxation of the bones complete, and their extremities as moveable as in a recent luxation from injury, the limb presenting to the eye of the almost frightened attendant an appearance of greater deformity and weakness than before the commencement of his well-intentioned but injudicious management." (p. 55.) In order to remedy lateral displacement of the tibia, Dr. Little attaches to his apparatus pads fixed to screws for the purpose of pressing the extremities of the tibia and of the femur in appropriate directions: and when the head of the tibia is thrown backwards the apparatus is so contrived as to constitute the pelvis as a fixed point, and make such extension from the ankle and the foot as will not only *extend* the entire limb " but elongate to such an extent the ligaments of the joint and other structures exterior to it, as will enable the tibia to rise into its position." (p. 55.) When the extremities of the bones are presumed to be sufficiently separated by the extension thus applied, pressure is so directed as to aid in placing them in apposition while the ankle is drawn inwards to remove any eversion of the foot that may exist. M. Duval's practice is in some respects very different: his apparatus is so contrived as to carefully avoid making any pressure on the knee; when the displacement of the head of the tibia is not very great he does not seem to make any attempt to remedy it; and when it is great, he still employs the ordinary extending apparatus; but when the joint has been nearly, or even completely, straightened, he places the patient for a few hours every day on an orthopedic bed, and, the trunk having been fixed, submits the leg to graduated parallel extension by means of a lace passing from the ankle over a pulley and attached to a spiral spring whose force can be regulated at pleasure. To judge both from the text of the cases recorded by Dr. Little and M. Duval and from the figures by which the cases are illustrated, the results obtained by the former practitioner are by much the more satisfactory and perfect.

When extension is completed, M. Duval makes his patients still wear the extending apparatus while walking, the foot-board being replaced by a leather gaiter; and the use of a stick, much more of crutches, is strictly prohibited, as calculated to discourage the patient from exertion and prolong weakness of the limb; after some time the apparatus is removed at night and replaced first every day, then every second day, and so on, at longer intervals, until it is finally discontinued, and then the treatment terminates. With Dr. Little, however, the third, and often the most difficult stage of the treatment now commences; the restoration, namely, of the motions of the joint, which is to be effected by passive movements, frictions, vapour-baths, and the occasional application of the extending apparatus, its action being reversed so as to flex the joint. Dr. Little permits exercise as soon as the joint is straightened, but generally the limb must be supported for weeks or even months by an iron on its outer side with a spring to assist the weak extensor muscles of the knee, and subsequently a lead knee-cap must be worn.

When in deformity resulting from disease of the hip-joint, the resistance arising from muscular contraction cannot be overcome by frictions and passive exercise of the limb, &c.; " an apparatus consisting of a circular bandage fastened to the pelvis, and sometimes to the shoulders, with a steel spring attached behind to the femur, in order to effect extension of the member, may be worn, and will, in a short time, sufficiently remove the contraction to permit the application of the entire sole to the ground." (Little, pp. 37-8.) Should this prove insufficient, dividing the contracted muscles offers the only chance of cure, but should be postponed for two or three years after the complete subsidence of the original disease, to avoid the risk of again exciting the original disease, but in the meantime the apparatus above mentioned should be worn to prevent aggravation of the deformity. The prognosis in contraction of the hip should be more guarded than in the case of other joints, because of the greater difficulty in ascertaining the precise amount of change the structures have undergone. Dr. Little gives five cases of anchylosis of the hip-joint, all of which were materially benefited by the operation except one, in which the deformity was so aggravated, and circumstances otherwise so unfavorable as to leave little to be hoped for. A brief abstract of the third case may illustrate the author's practice. A boy nine years and a half old, had the right hip contracted from the age of three, in consequence of a fall; "the part presents the ordinary appearance of false anchylosis. The knee is much drawn up towards the abdomen, and the thigh is rotated inwardly." (85.) Manipulations and frictions were employed without effect for five weeks, when Dr. Little divided the more prominent muscles; "viz., the tendons of rectus femoris, origin of pectineus, adductor longus, and part of adductor magnus." On the third day the punctures were cicatrized, and the thigh could be depressed about four inches; frictions and manipulations were resumed, and "two months after the operation he had abandoned the crutch, and walked comparatively well; the greater portion of remaining lameness depended on shortening of the limb, and some unnatural flexion, for which the hollowness of the loins compensated." (p. 86.) When, as often occurs, anchylosis of the hip is complicated with contraction of the corresponding knee and ankle, those parts will require a separate appropriate treatment.

We have said enough to convey a sufficient idea of Dr. Little's work, and of that portion of M. Duval's which treats of false anchylosis of the knee. Dr. Little also considers anchylosis of the other joints of the extremities, and of the lower jaw and vertebræ; but we shall not advert to those sections of his book further than to say that we wish he had considered the question of the applicability of tenotomy to the flexor tendons of the fingers at somewhat greater length, but we hope to deal with this subject on another occasion. Before taking leave of Dr. Little's book we must recommend it to our readers, and we trust it will contribute to awaken the attention of the profession *generally* to a class of deformities —those consequent on diseases of the joints—which long were considered incurable, and sometimes even regarded as favorable terminations of the formidable maladies in which they originated. To M. Duval, we believe, the credit is due of having first attempted to remove this opprobrium of surgery, and his present work shows how far and how well he has succeeded. Dr. Little has most successfully pursued the path thus opened and obtained results decidedly superior to those of his predecessor.

M. Dancel's pamphlet need not detain us long; the author's practice can be collected almost entirely from his title-page, which is copied at the head of this article, and the doctrine on which he founds that practice may be briefly explained. M. Dancel denies that the contracted muscles in angular anchylosis are really or organically shortened; however rigid, however permanently contracted muscles may be in consequence of any inflammatory disease of the joint, they are merely, he says, in a state of spasmodic contraction, which is a nervous affection, a spasm capable of being cured by compression and extension; tenotomy he grants will also destroy the spasm, but it is rarely necessary to recur to it; at least, the necessity of so doing arises much less frequently than is commonly supposed. M. Dancel adduces nine cases in support of his views, which we shall not analyse, as none of them really support his doctrine. Some of them, no doubt, are cases of anchylosis; of the knee, for example, after white swelling, cured by splints, bandages, &c., so applied as to effect gradual extension of the leg; but no one, not the most strenuous advocate of tenotomy, denies that this may be occasionally effected; on the contrary, where such means offer a prospect of cure, the tenotomists themselves employ them. Some others of M. Dancel's cases, and those to which he seems to attach most importance, cannot be considered cases of anchylosis; they were clearly cases of permanent muscular spasm arising from various causes, e. g. permanent muscular spasm from exposure to cold, and contraction of the biceps muscle consequent on venesection at the head of the arm. In such cases, however, we think M. Dancel's method a good one, and it is for this reason that we have noticed his work. His method is to powerfully compress the affected muscle by a roller applied round the limb so tightly that it cannot be borne for more than a few minutes; it is not indispensable to compress the entire muscles; acting on even a small portion of it will suspend the spasm: when the bandage is removed, the muscle is partially paralysed as it were, and can be completely or partly extended; splints, &c., are applied to maintain the degree of extension maintained, the greatest care being taken to compress (by bandaging) the muscle as forcibly as can be permanently borne. The mode of action of the very forcible compression first applied is easily understood; and so far M. Dancel's practice is, in fact, an extension to permanent spasm, of a method occasionally useful in intermittent spasm, or convulsions of the muscles of an extremity.

---

### ART. VIII.

*Medico-Chirurgical Transactions.* Vol. XXVI.—*London*, 1843. 8vo. Three Plates.

